# Adult attention deficit hyperactivity disorder symptom profiles and concurrent problems with alcohol and cannabis: sex differences in a representative, population survey

**DOI:** 10.1186/s12888-016-0746-4

**Published:** 2016-02-27

**Authors:** Nathan J. Kolla, Mark van der Maas, Maggie E. Toplak, Patricia G. Erickson, Robert E. Mann, Jane Seeley, Evelyn Vingilis

**Affiliations:** Centre for Addiction and Mental Health, Toronto, ON Canada; Department of Psychiatry, University of Toronto, Toronto, ON Canada; Department of Sociology, University of Toronto, Toronto, ON Canada; Department of Psychology, York University, Toronto, ON Canada; Dalla Lana School of Public Health, University of Toronto, Toronto, ON Canada; Department of Family Medicine, University of Western Ontario, London, ON Canada; CAMH, 250 College Street, Toronto, ON Canada

## Abstract

**Background:**

Adult attention deficit hyperactivity disorder (ADHD) shows a robust association with alcohol and cannabis misuse, and these relationships are expressed differently in males and females. Manifestation of specific ADHD symptom profiles, even in the absence of the full disorder, may also be related to problems with alcohol and cannabis, although these relationships have not been investigated in epidemiological studies. To address this question, we studied the sex-specific associations of ADHD symptomatology with problematic alcohol and cannabis use in a representative sample of adults aged 18 years and older residing in Ontario, Canada.

**Methods:**

Data were obtained from the Centre for Addiction and Mental Health Monitor, an ongoing cross-sectional telephone survey, between January 2011 and December 2013. Respondents (*n* = 5080) reported on current ADHD symptomatology, measured using the Adult ADHD Self-Report Version 1.1 Screener (ASRS-V1.1) and four additional items, and alcohol and cannabis use, which were measured using the Alcohol Use Disorders Identification Test (AUDIT) and the Alcohol, Smoking and Substance Involvement Screening Test (ASSIST), respectively. Logistic regression analyses were conducted in men and women to test the association of each ADHD symptom cluster (hyperactivity, inattentiveness, impulsivity) with problematic alcohol and cannabis use.

**Results:**

After controlling for age, education, and comorbid internalizing and externalizing psychopathology, hyperactive symptoms were associated with problematic alcohol use in both men and women and with problematic cannabis use in men. Impulsive symptoms were independently associated with problematic cannabis use in men. By contrast, inattentive symptomatology predicted problems with alcohol and cannabis only in women. In all models, age was negatively associated with substance misuse and externalizing behavior was positively correlated and the strongest predictor of hazardous alcohol and cannabis use.

**Conclusions:**

ADHD symptom expression in adulthood is related to concurrent hazardous use of alcohol and cannabis. Distinctive ADHD symptom profiles may confer increased risk for substance misuse in a sex-specific manner.

## Background

More individuals receive treatment for problematic alcohol and cannabis use than any other substance of abuse [1]. Links between alcohol or cannabis misuse and attention deficit hyperactivity disorder (ADHD), a neurodevelopmental illness comprising symptoms of inattention, hyperactivity, and impulsivity, have been identified in adults [2, 3]. Some reports have additionally detected relationships between ADHD symptom burden in adulthood and severity of substance misuse [4], suggesting that certain ADHD symptom profiles may influence expression of problematic alcohol and cannabis use in ADHD.

By contrast, only a handful of studies have tested the association of ADHD symptom domains with substance use measures in non-clinical samples of adults. This research is a vital line of inquiry, since subthreshold ADHD phenotypes are likely more common than the clinical disorder and may also be associated with hazardous alcohol or cannabis use. Two studies that investigated samples of non-treatment seeking college students reported relationships between ADHD symptom clusters and substance use outcomes. One found a positive association of inattentive symptoms with alcohol use and cannabis misuse that was independent of comorbid internalizing and externalizing psychopathology [5], while the other linked inattentive and hyperactive symptoms to greater cannabis use after controlling for conduct disorder and antisocial personality disorder [6]. While these studies describe an important connection between ADHD symptom domains and potentially hazardous substance use, results were likely influenced by self-selection bias and sampling of higher functioning populations [5, 6].

Epidemiologic study designs can improve upon these methodological limitations by providing estimates of key associations at a population level. To the best of our knowledge, only one cross-sectional, population-based study has analyzed ADHD symptom domains in relation to substance use outcomes [7]. After controlling for lifetime history of major depressive disorder, anxiety disorders, and conduct disorder, hyperactive-impulsive symptoms present by age 17 years in an adult population sample were associated with increased risk of lifetime alcohol and cannabis use disorder. Inattentive symptoms additionally predicted lifetime history of cannabis use disorder [7]. These results provide initial epidemiological evidence of a link between dimensional ADHD measures and substance misuse but do not answer the critical question of whether active ADHD symptomatology in adulthood predicts contemporaneous problems with alcohol or cannabis.

Another important gap in the literature relates to the paucity of information on potential sex differences associated with ADHD symptom expression and substance misuse. Most population studies that examined ADHD diagnosis or symptomatology and substance use measures controlled for sex in the adjusted analyses and did not report on any sex-specific relationships [7–10]. Understanding how sex may influence these relationships has clinical relevance, since recent evidence indicates that risk for alcohol and substance use disorders in ADHD may be greater in women [11]. However, it is presently unknown whether ADHD symptoms and substance misuse manifest differently in men and women at a population level. Hence, there is a pressing need for robust epidemiological data to better understand the manifestation of ADHD symptom cluster expression by substance use outcomes and sex. The objective of the present study was to investigate the association of active ADHD symptom domain expression with problematic alcohol and/or cannabis use by sex in a population-based, representative study of Canadian adults. We hypothesized that ADHD symptom expression would predict problematic substance misuse in both men and women even after controlling for age, education, internalizing symptoms, and externalizing symptoms and that these relationships would be expressed differentially by sex.

## Methods

### Participants and procedures

Telephone interviews were conducted over 36 months between January 2011 and December 2013 in a similar manner as previously reported [12]. Informed consent was obtained from the participants at the start of the telephone survey. Data were acquired from the Centre for Addiction and Mental Health (CAMH) Monitor, a cross-sectional, computer-assisted telephone survey (landlines and cellphones) of Ontario residents (18 years and older) conducted by CAMH, Toronto, Ontario, and administered by the Institute for Social Research at York University, Toronto, Ontario. For details, see [13]. Each monthly cycle of the survey utilized a two-stage probability sampling procedure. In the first stage, a random sample of telephone numbers was selected with equal probability from each regional stratum. In the second stage, one respondent from each household was selected based on the following criteria: 1) age 18 years or older; 2) most recent birthday from the date of the telephone interview; and 3) ability to complete the interview in English. Response rates based on estimated eligibility of the sample averaged 50 %. No information was available for the non-participants. All study components were approved by the Research Ethics Boards of CAMH, York University, and the University of Western Ontario in London, Ontario.

Results used in the analyses were based on “valid” responses to survey questions. That is, responses such as “don’t know” or refusals were treated as missing data and excluded from analyses. Overall, 994 individuals were excluded from the analyses due to missing data. The final sample included 5080 individuals who completed all study measures. Altogether, there were 2066 males (40.7 %) and 3014 females (59.3 %). Participant ages ranged from 18 to 97 years with a mean age of 54.4 ± 16.5 years. Regarding education, 31.8 % completed high school or less, 36.6 % completed some or all requirements for community college or a technical diploma, and 31.6 % completed some or all requirements for a Bachelor’s degree or higher.

### Measures

#### Adult ADHD Self-Report Version 1.1 Screener (ASRS-V1.1)

The ASRS-V1.1 [14] is derived from symptoms of ADHD as identified in the Diagnostic and Statistical Manual of Mental Disorders, Fourth Edition (DSM-IV; [15]). Among the 18 DSM-IV symptoms of ADHD, 6 were found to be predictive of ADHD as determined by clinical assessment, and these items comprise the ASRS-V1.1. Four of the items relate to symptoms of inattention and two index hyperactivity symptoms. The ASRS-V1.1 was selected over the full version of the ASRS, because we required parsimony given the length of the telephone survey. Moreover, the ASRS-V1.1 is more sensitive and specific and offers better classification accuracy than the ASRS [14, 16, 17]. For the present study, three additional questions tapping impulsivity symptoms of ADHD (e.g., finishing others’ sentences, difficulty waiting turn, and interrupting others) outlined in the most recent edition of the DSM (DSM-5 [18]) were included to provide an index of impulsivity. An additional item relating to hyperactivity (e.g., difficulty remaining seated) was included to provide an equal number of impulsive and hyperactivity symptoms. These items were drawn from the full version of the ASRS. Participants were required to rate each item using a 5-point, Likert-type scale with higher scores indicative of more severe symptoms. We selected these items, because a previous factor analysis [19] showed the presence of a general factor within an adult sample that used these same 10 items from the ASRS as an ADHD screener. Furthermore, a three-factor model that separated impulsivity and hyperactivity was found to summarize the data better than a combined hyperactivity/impulsivity factor.

The standardized Cronbach’s alpha for the full 10-item scale was 0.80, indicating good internal consistency [20]. The Cronbach’s alpha coefficients for the inattention, impulsivity, and hyperactivity subscales were 0.77, 0.62, and 0.54, respectively. The lower internal consistency of the inattention and impulsivity subscales was not unexpected given that the subscales comprised four and three items, respectively.

### Alcohol Use Disorders Identification Test (AUDIT)

The AUDIT is an extensively validated 10-item, Likert-type screening instrument that was developed by the World Health Organization (WHO) and designed to identify individuals presenting less severe alcohol problems [21, 22]. A score of eight or greater on the AUDIT is indicative of hazardous alcohol use. The use of a cut-off score of 8+ as a positive screen for both males and females has been used in Ontario and Canadian national surveys [23, 24].

### Alcohol, Smoking and Substance Involvement Screening Test (ASSIST)

The cannabis subscale of the ASSIST is a 6-item screening instrument that assesses risk of experiencing health, financial, legal, social, and relationship problems associated with cannabis use [25]. A score of four or more is indicative of moderate or high risk of problematic use [26]. The ASSIST cannabis subscale showed overall good test-retest reliability in the original study.

### General Health Questionnaire (GHQ12)

The GHQ12 is a 12-item screening instrument that captures current symptoms of depression, anxiety, and social functioning [27]. Items are rated on a 4-point, Likert-type scale. Higher scores reflect greater psychological distress, and a score of 3 or greater denotes a positive screen. The GHQ12 was developed for use in adult, non-clinical populations and demonstrates strong psychometric properties [28].

### Conduct disorder symptoms

The Antisocial Personality Disorder Scale from the Mini-International Neuropsychiatric Interview (MINI-APD) is a 12-item, dichotomous scale that assesses delinquent behavior and provided a measure of externalizing psychopathology [29]. One item was excluded from the MINI-APD (forced someone to have sex before age 15 years) as required by our institutional review board. Externalizing psychopathology for the current study was defined as one or more delinquent behaviors present before age 15 years.

### Demographic variables

Since increasing age relates to a decline in ADHD symptoms [30] and lower socioeconomic status has been linked to an ADHD diagnosis [31], we included age and education in the analyses.

### Statistical analysis

For each of the variables included in the analyses, comparisons were made between men and women in the sample. Continuous variables were compared using independent sample *t*-tests or the Mann-Whitney U test for those variables not following a normal distribution. Dichotomous variables were compared using chi-square tests. A series of logistic regressions were then applied to estimate the associations of problematic alcohol or cannabis use with ADHD symptom domains. Logistic regression was chosen, because we were interested in testing the relationship between ADHD symptom severity and alcohol and/or cannabis misuse as categorical outcomes. Forced entry logistic regression was employed, given that the models were theoretically-driven and there were relatively few predictor variables. For each dependent variable (problematic alcohol use or problematic cannabis use), three separate models were constructed, one for each ADHD symptom cluster (hyperactive, impulsive, and inattentive symptoms) indexed by the ASRS-V1.1 screener and supplementary items. We considered entering inattention and hyperactivity/impulsivity symptom domains simultaneously in the regression analyses; however, we were interested in understanding the separate contributions of inattention, hyperactivity, and impulsivity symptom profiles in the current study. The specific inattention and hyperactivity/impulsivity factors, after the common variance was accounted for by a general factor model, have been interpreted as residual and unclear for interpretation [19]. Thus, we opted to enter the domains of inattention, hyperactivity, and impulsivity as separate predictors in each regression analysis. To achieve our study objective and determine whether differences emerged between sexes, each regression model was run separately for men and women. Odds ratios with 95 % confidence intervals were generated from these regression analyses. Comparisons between models were made using the Akaike Information Criterion (AIC). The variance inflation factor (VIF) was examined for each model to inspect for high inter-correlations among independent variables. The VIF was less than 1.3 for each model, suggesting that estimates were not significantly affected by collinearity [32]. All analyses were completed using the R project for statistical computing [33].

## Results

### Sample characteristics

Comparisons between men and women on demographic variables, measures of internalizing and externalizing psychopathology, substance misuse, and ADHD symptom cluster scores are presented in Table 1. The two groups were similar in age and education level. Men endorsed more externalizing psychopathology and less psychological distress than women. Women reported greater active inattentive and impulsive symptomatology, whereas males reported higher rates of problematic alcohol and cannabis use.

**Table 1:** Demographic and clinical variables

	Women	Men	Test statistic *p* value	
Demographics				
Age^a^	54.0 ± 16.1	53.4 ± 16.2	−1.24^c^	0.223
Postsecondary education^b^	69.0	67.2	1.82^d^	0.157
ADHD Symptomatology				
Inattention^a^	3.1 ± 2.9	2.8 ± 2.7	3.68^e^	0.000
Hyperactivity^a^	2.1 ± 2.2	2.1 ± 2.2	0.64^e^	0.534
Impulsivity^a^	2.7 ± 2.1	2.4 ± 2.0	4.74^c^	0.000
Substance Abuse Measures				
ASSIST^b^	2.7	6.1	34.77^d^	0.000
AUDIT^b^	6.0	17.7	176.21^d^	0.000
Psychiatric Symptom Measures				
GHQ-12^b^	15.4	11.0	20.07^d^	0.000
Conduct disorder symptoms^b^	15.7	25.1	90.53^d^	0.000

*ASSIST* Alcohol, Smoking and Substance Involving Screening Test, *AUDIT* Alcohol Use Disorders Identification Test, *GHQ-12* General Health Questionnaire

^a^value expressed as mean ± standard deviation; ^b^value expressed as percentage; ^c^ *t*-test; ^d^chi-square; ^e^Mann-Whitney U

### Regression analyses

As depicted in Table 2, problematic drinking was related to differential expression of ADHD symptom domains by sex. For both sexes, hyperactive symptoms were positively associated with problematic alcohol use and impulsive symptomatology also predicted alcohol misuse in men. In women, but not men, severity of inattentive symptoms additionally predicted problematic alcohol use. In all models, externalizing psychopathology emerged as the strongest predictor of alcohol misuse. Age was also negatively correlated with problematic alcohol use in all models. No relationship was detected between active internalizing symptoms and problematic drinking, controlling for age, education, externalizing psychopathology, and ADHD symptom domains.

**Table 2:** Logistic regression for positive AUDIT screen divided by sex

	Women			Men		
	Odds ratio	Critical intervals	*p* value	Odds ratio	Critical intervals	*p* value
Model 1						
Intercept	0.292	0.160–0.525	0.000	0.545	0.361–0.820	0.004
Hyperactivity	1.132	1.063–1.205	0.000	1.073	1.023–1.124	0.003
Age	0.961	0.951–0.971	0.000	0.971	0.965–0.978	0.000
Conduct Disorder Symptoms	1.621	1.120–2.312	0.009	1.664	1.330–2.079	0.000
Postsecondary Education	0.882	0.625–1.261	0.480	1.217	0.970–1.535	0.093
Psychological Distress	1.038	0.698–1.513	0.851	1.092	0.806–1.465	0.564
Akaike Information Criterion	1254.9			2224.5		
Model 2						
Intercept	0.402	0.228–0.697	0.001	0.678	0.466–0.982	0.041
Inattention	1.060	1.007–1.115	0.025	1.029	0.992–1.068	0.128
Age	0.958	0.948–0.968	0.000	0.969	0.962–0.975	0.000
Conduct Disorder Symptoms	1.632	1.125–2.333	0.008	1.745	1.400–2.172	0.000
Postsecondary Education	0.814	0.578–1.161	0.245	1.166	0.931–1.468	0.185
Psychological Distress	1.050	0.699–1.550	0.808	1.099	0.804–1.489	0.550
Akaike Information Criterion	1264.3			2231.0		
Model 3						
Intercept	0.456	0.257–0.800	0.007	0.600	0.413–0.867	0.007
Impulsivity	1.021	0.953–1.091	0.555	1.091	1.039–1.145	0.000
Age	0.957	0.947–0.967	0.000	0.969	0.962–0.975	0.000
Conduct Disorder Symptoms	1.707	1.179–2.436	0.004	1.652	1.321–2.062	0.000
Postsecondary Education	0.833	0.593–1.188	0.303	1.151	0.918–1.451	0.227
Psychological Distress	1.196	0.810–1.731	0.355	1.093	0.808–1.466	0.556
Akaike Information Criterion	1269.5			2221.5		

Table 3 highlights the relationship between ADHD symptom clusters and problematic cannabis use in men and women. Results revealed that only inattentive symptoms predicted problematic cannabis use in women, whereas hyperactive and impulsive symptoms, but not inattentive symptomatology, predicted cannabis misuse in men. Similar to the regressions that tested problematic alcohol use as the dependent variable, age and externalizing psychopathology were negatively and positively associated with problematic cannabis use, respectively. Externalizing psychopathology was the strongest predictor of cannabis misuse in all models. However, in contrast to the previous regression models, current internalizing psychopathology predicted problematic cannabis use, controlling for ADHD symptom domains, externalizing psychopathology, and demographic variables in five of the six analyses.

**Table 3:** Logistic regression for positive ASSIST screen divided by sex

	Women			Men		
	Odds ratio	Critical intervals	*p* value	Odds ratio	Critical intervals	*p* value
Model 1						
Intercept	0.208	0.091–0.463	0.000	0.494	0.276–0.878	0.017
Hyperactivity	1.060	0.976–1.148	0.162	1.083	1.014–1.156	0.017
Age	0.938	0.922–0.953	0.000	0.952	0.941–0.963	0.000
Conduct Disorder Symptoms	4.024	2.642–6.101	0.000	2.594	1.894–3.553	0.000
Postsecondary Education	1.327	0.818–2.236	0.269	0.525	0.384–0.719	0.000
Psychological Distress	1.938	1.232–3.007	0.004	1.663	1.120–2.434	0.010
Akaike Information Criterion	785.7			1116.3		
Model 2						
Intercept	0.185	0.084–0.392	0.000	0.727	0.435–1.207	0.220
Inattention	1.129	1.060–1.203	0.000	1.002	0.950–1.055	0.937
Age	0.936	0.920–0.951	0.000	0.948	0.938–0.959	0.000
Conduct Disorder Symptoms	3.583	2.328–5.484	0.000	2.768	2.031–3.775	0.000
Postsecondary Education	1.213	0.748–2.039	0.448	0.499	0.366–0.682	0.000
Psychological Distress	1.538	0.956–2.435	0.070	1.819	1.204–2.712	0.004
Akaike Information Criterion	777.3			1120.2		
Model 3						
Intercept	0.230	0.104–0.491	0.000	0.599	0.357–0.999	0.051
Impulsivity	1.052	0.966–1.142	0.234	1.076	1.003–1.153	0.040
Age	0.936	0.920–0.951	0.000	0.949	0.939–0.959	0.000
Conduct Disorder Symptoms	3.974	2.600–6.046	0.000	2.617	1.911–3.583	0.000
Postsecondary Education	1.260	0.780–2.112	0.361	0.494	0.362–0.675	0.000
Psychological Distress	1.980	1.262–3.062	0.002	1.707	1.151–2.493	0.007
Akaike Information Criterion	785.1			1121.3		

## Discussion

As far as we are aware, this is the first population-based study of adults to examine the association of active hyperactive, impulsive, and inattentive symptomatology with problematic alcohol and cannabis use. Accumulating evidence from genetic studies suggests that conceptualization of ADHD symptomatology as a continuum of quantitative traits may be preferable to categorical approaches [34]. Accordingly, the use of dimensional measures that reflected active ADHD symptom burden and instruments that captured current substance use problems and psychiatric symptoms allowed us to draw inferences about how adult expression of ADHD symptom profiles may relate to concurrent problems with alcohol or cannabis. We detected modest associations between ADHD symptom clusters and substance misuse that were expressed differently by sex. In keeping with proposals advocating for symptom-based approaches to researching psychiatric illness [35], these results highlight the clinical importance of ADHD symptom expression in predicting problematic substance use and suggest that interventions targeting substance misuse in ADHD could also be directed toward subclinical ADHD phenotypes associated with specific symptom profiles.

Our results reveal that hyperactive, impulsive, and conduct disorder symptoms all predicted problematic alcohol and cannabis use in males, which agrees with previous research linking markers of behavioral disinhibition to risky alcohol and cannabis use in non-clinical samples composed mainly of men [36, 37]. Taken together, these findings accord well with a model that organizes antisocial behaviors, substance misuse, and impulsive traits along a spectrum of externalizing psychopathology [38], for which there is growing evidence of a common genetic liability [39]. Since dimensional measures of high ADHD symptomatology show significant heritability [40], one explanation for these findings is that a constellation of early externalizing behaviors and high adult hyperactive/impulsive symptom burden may represent an intermediate phenotype of adult problematic substance use. Support for this hypothesis comes from twin research reporting a shared genetic influence of adolescent hyperactive/impulsive ADHD and conduct disorder on alcohol dependence in adult males [41]. Population-based investigations that examined the genetic mechanisms of high impulsive and hyperactive symptomatology in adults with hazardous substance use could advance our understanding of potential subclinical ADHD phenotypes linked to adverse health outcomes.

Among the three ADHD symptom clusters, hyperactivity showed the strongest relationship with problematic alcohol use in females. This result is broadly consistent with results from a longitudinal design that detected a relationship between hyperactive symptoms assessed during childhood and indicators of alcohol misuse among adult females [42]. Additional study results from the present investigation highlight a sex-specific association of inattentive symptomatology with alcohol and cannabis misuse that was present only in females. Prior research connects inattentive ADHD symptomatology to cannabis and alcohol misuse [5, 43, 44], and one interpretation of our findings is that in general adult populations, the ADHD symptom domain of inattentiveness is more relevant to expression of risky alcohol and cannabis use in females compared with males. Since inattentive symptoms in ADHD exhibit a dose-dependent relationship with several forms of neurocognitive impairment [45] and are associated with poorer occupational functioning [46], manifestation of high inattentive symptomatology may relate to several markers of vulnerability that increase overall risk of problematic substance use in females. These associations could be mediated by common genetic underpinnings. For example, a genetic variant of the dopamine transporter gene, a molecular target of interest in ADHD, was found in one study to be more common in women with high inattentive symptoms [47], and this same genotype has also been linked to structural and functional brain changes in cannabis use disorders [48] and processing of alcohol cues [49].

It is notable that externalizing psychopathology emerged as the strongest predictor of alcohol and cannabis misuse in every model tested, although relationships between ADHD symptomatology and substance misuse still persisted after controlling for conduct disorder symptoms. The associations between externalizing psychopathology and hazardous cannabis use were particularly robust. There is some debate in the literature as to whether associations between ADHD and substance misuse are mediated exclusively by conduct-disordered behaviors. For example, one longitudinal study of a non-clinical sample examined childhood behaviors in relation to adult substance use disorders and reported that oppositional but not ADHD symptom clusters predicted cannabis use disorders in adulthood [50]. By contrast, other studies have found that both ADHD and externalizing behaviors assessed in adulthood predict cannabis misuse [5]. Discrepant results likely relate to the variable developmental contexts in which ADHD symptoms were measured [5] and differences in sample characteristics and operationalization of externalizing psychopathology.

An important clinical implication of our study is the potential for enhanced detection of problematic alcohol and/or cannabis use through identification of high ADHD symptom expression. Several reasons point to a growing awareness of adult ADHD in the general public [51], and front line clinicians will likely come in contact with increasing numbers of patients, including parents of children with ADHD, who query the disorder in themselves. Many of these individuals will not meet diagnostic criteria for ADHD but may still endorse high levels of ADHD symptomatology associated with problematic health behaviors. Since patients typically under-report or do not disclose their substance use [52], screening for hazardous drug or alcohol use in younger adult age groups who present subthreshold ADHD could represent a relatively simple and efficient means of identifying a subset of individuals at high risk for substance misuse. Furthermore, given evidence that treatment of ADHD may lead to a reduction in substance use [53], our results provide incentive for the development of age-appropriate interventions that could potentially address problematic substance use in specific subthreshold ADHD profiles.

We note several limitations of the present investigation common to most epidemiological surveys. First, given the cross-sectional study design, we are unable to make inferences about the direction of the relationship between ADHD symptom expression and problematic substance use. On the other hand, longitudinal studies have generally found that childhood ADHD predicts development of alcohol and cannabis use disorders in adulthood [54], suggesting a similar temporal ordering of high ADHD symptom expression and problematic substance use. Second, data were self-reported and did not include verification from informants that is typically required to make a clinical diagnosis of ADHD. However, since the purpose of the study was to assess ADHD symptomatology, as opposed to the clinical disorder, and strong agreement has been demonstrated between self- and observer-reported severity of adult ADHD symptoms [55], this aspect of the study design offered an efficient strategy to address our main research questions. Third, although our response rate of over 50 % compares favorably with other telephone surveys, and data were weighted to reflect a representative sample of adults from the third to tenth decades of life, it is still possible that sampling bias was present. As the participant age range was quite broad, it is also possible that the observed associations were not representative of all age groups, given that age was a significant predictor for each model. Fourth, our results may have been affected by response bias, as some evidence suggests that subjects may under-report or provide more favorable information about their alcohol and substance use when data are obtained from telephone interviews compared with anonymous surveys [56, 57]. Finally, the possibility of selection bias in our study cannot be overlooked, especially since some reports indicate that individuals with more severe ADHD and alcohol use disorders may be less likely to participate in studies [58, 59], although other research has found that markers of cannabis use severity do not influence study participation [60].

## Conclusions

In summary, our findings highlight a previously unreported nexus between active ADHD symptom cluster expression and problematic alcohol and/or cannabis use in a general adult population. These observations are of interest clinically, because they show that specific ADHD symptom profiles, even without evidence of the clinical disorder, are still associated with adverse health outcomes. Associations between ADHD symptom expression and hazardous substance use manifested differently in males and females, suggesting that sex effects are critical to understanding these relationships. Our results merit further study of subthreshold ADHD phenotypes to determine whether clinical intervention leads to improved health outcomes.
